# Pituitary metastases from papillary carcinoma of thyroid: a case report and literature review

**DOI:** 10.1530/EDM-13-0024

**Published:** 2013-08-30

**Authors:** Viral Chikani, Duncan Lambie, Anthony Russell

**Affiliations:** 1 Department of Diabetes and EndocrinologyThe Princess Alexandra Hospital199 Ipswich Road, Woolloongabba, Queensland, 4102Australia; 2 Department of Anatomical PathologyThe Princess Alexandra Hospital199 Ipswich Road, Woolloongabba, Queensland, 4102Australia

## Abstract

Metastases to the pituitary gland are an uncommon complication of thyroid cancer. They resemble pituitary neoplasms posing a diagnostic challenge. We present a case of an aggressive non-radioiodine avid papillary thyroid cancer with recurrent pituitary metastases and a review of the literature. A 70-year-old woman with a history of papillary thyroid cancer and bony metastases presented with symptoms of hypoadrenalism and peripheral vision loss. Magnetic resonance imaging showed a large pituitary mass impinging on the optic chiasm. She underwent transsphenoidal resection followed by ^131^I ablation. Post-therapy scintigraphy showed no iodine uptake in the sellar region or bony metastases. Histology of the pituitary mass confirmed metastatic papillary thyroid cancer. Fifteen months later, she had a recurrence of pituitary metastases affecting her vision. This was resected and followed with external beam radiotherapy. Over 2 years, the pituitary metastases increased in size and required two further operations. Radioactive iodine was not considered due to poor response in the past. Progressively, she developed a left-sided III and IV cranial nerve palsy and permanent bitemporal hemianopia. There was a rapid decline in the patient's health with further imaging revealing new lung and bony metastases, and she eventually died 8 months later. To our knowledge, this is the first case of pituitary metastases from a radioiodine-resistant papillary thyroid cancer. Radioiodine-resistant metastatic thyroid cancer may exhibit rapid aggressive growth and remain poorly responsive to the currently available treatment.

**Learning points:**

- Differentiated thyroid cancer (DTC) has an excellent prognosis with <5% of the cases presenting with distant metastases, usually to lung and bone.
- Metastasis to the pituitary is a rare complication of DTC.
- The diagnosis of pituitary insufficiency secondary to pituitary metastases from DTC may be delayed due to the non-specific systemic symptoms of underlying malignancy and TSH suppression therapy for thyroid cancer.
- The imaging characteristics of metastases to the pituitary may be similar to non-functioning pituitary adenoma.
- Radioiodine refractory metastatic thyroid cancer has significantly lower survival rates compared with radioactive iodine-avid metastases due to limited therapeutic options.

## Background

Pituitary metastases are a rare complication of differentiated thyroid cancer (DTC) (1). Patients often present with symptoms of pituitary insufficiency or symptoms related to mass effects. However, due to the non-specific systemic symptoms of underlying malignancy and TSH suppression therapy for thyroid cancer, the diagnosis of pituitary insufficiency is often delayed. Very little information is available on the imaging characteristics of pituitary metastases.

Here, we report a case of pituitary metastases from a radioiodine-resistant papillary thyroid cancer. This case report describes in detail the magnetic resonance imaging (MRI) characteristics and the histopathological findings of pituitary metastases from papillary thyroid cancer and provides an understanding of the natural history and prognosis of this rare disease.

## Case presentation, management and outcome

A 70-year-old woman was under the endocrine unit since 2003 for the management and ongoing surveillance of metastatic papillary thyroid carcinoma. She initially presented in 1988 with a metastatic deposit of follicular variant of a papillary carcinoma of the thyroid in the manubrium that was iodine avid. She underwent a total thyroidectomy with partial resection of the manubrium followed by radioactive iodine ablation. Over a period of 15 years, she received a further five courses of ^131^I ablation, manubriumectomy in 2003 for a relapse of metastatic disease and radiotherapy to T1 vertebral metastases in 2007.

**Figure 1:**
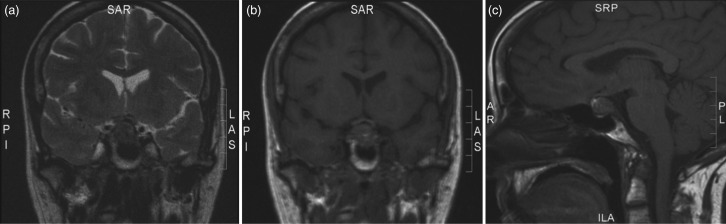
2006 MRI pituitary: (a) coronal T2WI, (b) coronal T1WI and (c) sagittal T1WI respectively.

**Figure 2:**
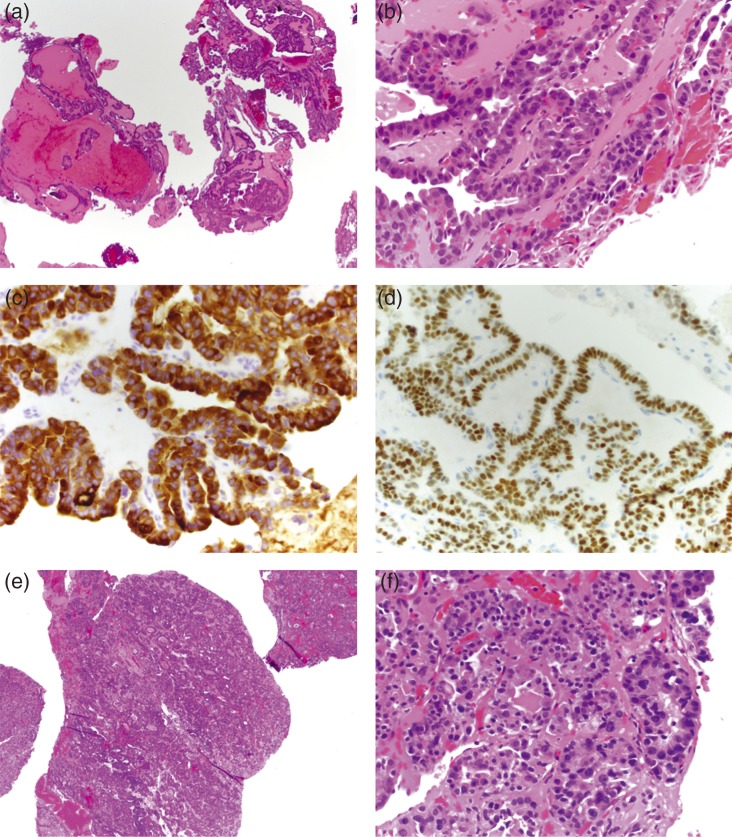
(Panels a-d) Histopathology of pituitary metastasis at diagnosis: (a) pituitary metastasis biopsy displaying well-formed papillae and cystic change (H&E 40× original magnification); (b) nuclear features of papillary thyroid carcinoma are present including some nuclear overlapping, nuclear grooves and ‘dusty’ chromatin (H&E 400× original magnification); (c) strong and diffuse reactivity for thyroglobulin (400× original magnification); and (d) diffuse nuclear reactivity for thyroid transcription factor-1 (400× original magnification). (Panels e-f) Histopathology of recurrence in pituitary metastasis 4 years later: (e) it is showing less well-developed architecture with cribriform and follicular patterns (H&E 40× original magnification) and (f) it is showing marked variation in nuclear size, hyperchromasia and membrane irregularities (H&E 400× original magnification).

In 2006, during an outpatient visit, she relayed a 12-week history of 7 kg weight loss, nausea and vomiting. She was admitted to hospital for investigation of these symptoms, including computerised tomography (CT) of the abdomen, gastroscopy and colonoscopy with no apparent cause found. Subsequently, on resolution of these symptoms after receiving an intra-articular steroid injection for shoulder pain, it was discovered that she was hypocortisolaemic with a morning cortisol of 133 nmol/l and an abnormal short synacthen test (Table 1). Further investigations revealed mild hyperprolactinaemia and hypogonadotrophic hypogonadism. The TSH had been ‘suppressed’ from thyroid hormone treatment for metastatic thyroid cancer. Cortisone acetate was commenced with improvement of symptoms. Computerised perimetry showed a superior bitemporal quadrantanopia. MRI of the pituitary (Fig. 1) revealed a 1.5 cm mass lifting and thinning the optic chiasm with irregular signal intensity on T1 and mixed intermediate and low T2 signal. She underwent transsphenoidal resection and debulking of the pituitary mass. Histopathology and immunohistochemistry confirmed metastatic papillary thyroid carcinoma of the pituitary gland (Fig. 2A). Following this, she was treated with recombinant TSH and given ^131^I therapy. Unfortunately, her post-therapy scan and single-photon emission computed tomography (SPECT) did not show any activity in the pituitary fossa region or the thoracic spine, suggesting poor accumulation of iodine and thus ^131^I resistance. Her visual field defects improved following the surgery.

**Table 1:** Baseline anterior pituitary function tests preoperatively

**Tests** (units)	**Results**	**Reference range**
GH (μg/l)	0.2	<8.0
IGF1 (nmol/l)	7	7–30
FSH (U/l)	1.2	17–115
LH (U/l)	<0.2	11–59
Oestradiol (pmol/l)	33	<100
Prolactin (mU/l)	1660	58–416
ACTH (ng/l)	<10	10–50
Cortisol (nmol/l)	133	200–700
TSH (mU/l)	<0.05^a^	0.3–5.0
Free T_4_ (pmol/l)	21	9–23
Free tri-iodothyronine (T_3_; pmol/l)	4.8	3.0–5.5
Free alpha glycoprotein subunit (U/l)	<0.1	<2.0
Short synacthen test: cortisol (nmol/l)		
Time 0	20	220–660
+30 min	180	
+60 min	240	>550

IGF1, insulin-like growth factor 1.

a During thyroxine (T_4_) treatment.

**Figure 3:**
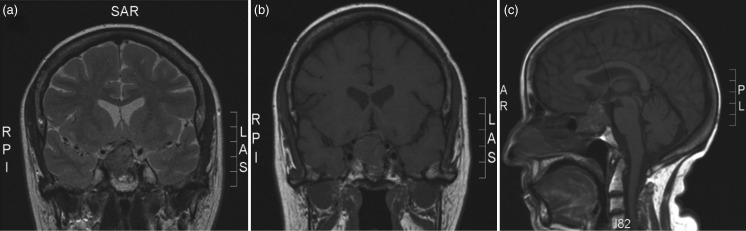
2008 MRI pituitary: (a) coronal T2WI, (b) coronal T1WI and (c) sagittal T1WI respectively.

Fifteen months post surgery, she represented with headaches. Repeat computerised perimetry showed worsening bitemporal field defects. Repeat MRI scan of the pituitary (Fig. 3) demonstrated a large 26×30×24 mm contrast enhancing mass with an irregular border centred within the pituitary fossa and extending into the sphenoid sinus and superiorly to abut and displace the optic chiasm. She underwent a second transsphenoidal resection and extensive debulking of the tumour, with histology again confirming metastatic papillary thyroid carcinoma. Surgery was followed by 50 Gy/25 fractions of external beam radiotherapy to the pituitary field. Radioactive iodine was not considered due to lack of previous uptake and to deterioration in her renal function over the last few years.

**Figure 4:**
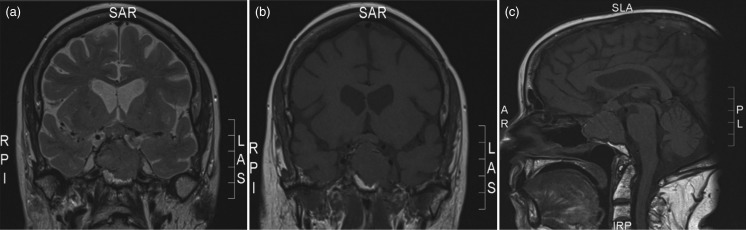
2011 MRI pituitary: (a) coronal T2WI, (b) coronal T1WI and (c) sagittal T1WI respectively.

She failed to achieve an adequate response from her radiotherapy, and over the next 3 years, the pituitary metastasis increased in its supra-sellar extent (Fig. 4). Computerised perimetry showed variable bitemporal hemianopia, which now crossed the midline and the ocular coherent tomography looking at the retinal nerve fibre layer showed temporal thinning. She underwent a third transsphenoidal debulking of her pituitary metastases, which showed an increase in tumour grading (Fig. 2B). Unfortunately, it markedly increased in size within a couple of months of surgery and the patient developed a left-sided ptosis and further deterioration of vision. In view of her threatened vision, she underwent a fourth debulking of the pituitary metastases, which was performed via transcranial approach. Post surgery, her vision deteriorated further with development of left IV cranial nerve palsy. On restaging imaging, she was found to have new pulmonary and bony metastases. She was assessed for a multicentre, randomised, double-blind, placebo-controlled, phase III trial of a targeted therapy, but she did not meet the criteria due to her poor Eastern Cooperative Oncology Group (ECOG) performance status, recurrent episodes of epistaxis and severe anaemia requiring blood transfusion with high clinical suspicion of occult upper gastrointestinal bleed. There was a progressive decline in vision and physical condition. She died 8 months after her last pituitary surgery.

## Discussion

Pituitary metastases are relatively uncommon, representing 1% of all sellar neoplasms from surgical and 5% in autopsy series (1). The most common metastatic malignancies to the pituitary gland are lung in men and breast in women (1). Thyroid cancer is an uncommon cause of pituitary/sellar metastases and, to date, where histological classification has been reported, only four cases of papillary thyroid cancer have been reported (Table 2).

**Table 2:** Characteristics of six published cases of pituitary metastases from papillary thyroid cancer

**References**	**Clinical presentation**	**Other metastases**	**Radiological features**	**Histological types**	**Treatment**	**Outcome**
(2)	Hypopituitarism	Lungs, bones	MRI: 1.2 cm homogeneously enhancing mass	Not available	^131^I ablation and chemotherapy	Long-term follow-up not reported
(2)	Diplopia and ptosis, hypopituitarism, vision loss	Lungs, right orbit	MRI: large mass involving cavernous sinus and pituitary fossa	Papillary thyroid cancer	Stereotactic radiotherapy and ^131^I ablation	Death within 12 months of diagnosis of pituitary metastases
(8)	Hypopituitarism	Mediastinum, bone	CT: large intrasellar pituitary tumour	Papillary thyroid cancer	Transsphenoidal surgery and ^131^I ablation	Died of massive intrathoracic haemorrhage
(9)	Seizure and visual disturbance	None	MRI: pituitary mass	Papillary thyroid cancer	Resection followed by ^131^I ablation	At 3-year follow-up, no evidence of recurrence
(10)	Visual disturbance, cranial nerve III, IV and V palsy	None	Skull radiograph: destruction of the floor of sella turcica	Not available	Radiotherapy and ^131^I ablation	At 20 months of follow-up, patient remained stable
(4)	Hemianopsia, diabetes insipidus, amenorrhoea	Lung	Not available	Papillary thyroid cancer	Transsphenoidal surgery	Long-term follow-up not reported

Owing to a high prevalence of pituitary tumours, diagnosis of pituitary metastases can be challenging. Non-specific systemic symptoms (fatigue, nausea and weight loss) of underlying malignancy and TSH suppression therapy in case of thyroid cancer often delay the diagnosis of hypopituitarism caused by pituitary metastases (2).

High-resolution CT and MRI are sensitive imaging modalities but lack specificity. The MRI of pituitary metastases demonstrates an iso-intense or hypo-intense mass on T1 with a hyper-intense signal on T2-weighted image (1). Mayr *et al*. (3) reported that seven of nine pituitary metastases were iso-intense on both T1- and T2-weighted images. Of six published cases of papillary thyroid cancer metastatic to pituitary, only three underwent MRI pituitary; however, detailed descriptions of signal characteristics are not available (see Table 1). MRI of pituitary in our patient revealed a mixed signal in both T1- and T2-weighted images.

Rapid aggressive tumour growth and infiltration of adjacent tissues favour the diagnosis of pituitary metastases. Pituitary metastases from thyroid cancer usually present with symptoms of mass effects like headache, visual loss or signs of other cranial nerve palsy due to supra-sellar extension with optic chiasm compression or cavernous sinus invasion respectively.

Owing to the direct arterial supply, metastases to the posterior lobe of the pituitary gland are more common. The most common symptom reported in the literature is diabetes insipidus in up to 45% of the cases (1). The incidence of diabetes insipidus in DTC seems much lower and so far only one conclusive case has been reported (4).

The overall 10-year disease-specific survival in DTC is 85% and it drops to 30–55% in radioactive iodine-avid metastases and 10–18% in non-radioactive iodine-avid metastatic disease (5). Standard treatment for DTC includes surgery, radioactive iodine and TSH suppression therapy. When thyroid cancers lose differentiated phenotype, they may not uptake the radioiodine and become ^131^I resistant. Radioiodine refractory metastatic thyroid cancer can be managed with thyroid hormone suppression therapy, external beam radiotherapy or chemotherapy (6). However, cytotoxic chemotherapy for metastatic thyroid cancer has a very limited role due to a poor response rate and significant side effects. The most studied chemotherapeutic agent is doxorubicin, which is used alone or in combination with cisplatin. Less than 25% of patients with DTC respond to standard chemotherapy and achieve only a partial response lasting no more than a few months (6).

Currently, there are a number of trials underway evaluating the role of therapies targeting specific molecules involved in the molecular and cellular pathogenesis of thyroid cancer. These therapies mainly target angiogenesis, tumour cell growth and apoptotic pathways, and oncogenic signalling pathways (6). In preliminary trials, partial responses are reported in ∼15–30% of patients and none of them has shown complete response.

The role of various redifferentiating agents enhancing iodine uptake by modulating thyroid gene expression to improve the efficacy of ^131^I ablation in non-radioiodine avid tumours has been well described in the literature and results from *in vitro* studies are promising; however, further studies are needed (7).

In conclusion, pituitary metastases are a rare complication of metastatic DTC. These metastases are aggressive, invasive and large, causing mass symptoms and profound hypopituitarism. These secondary malignancies, when no longer iodine avid, pose a very poor prognosis. The efficacy of other treatment options including external irradiation and chemotherapy is low. Targeted therapies may hold promise to deliver better outcomes for non-radioiodine avid metastatic thyroid disease.

## Patient consent

The consent form has not been obtained because the patient is dead and the information provided in this report is unidentifiable.

## Author contribution statement

V Chikani was involved in the patient's care as a registrar and he prepared the manuscript and reviewed the literature. D Lambie reported the histopathology slides. A Russell was the physician responsible for the patient and he reviewed and edited the manuscript.
